# Effects of cold spray on thirst, frequency of oral care, and pain of general surgery intensive care unit patients

**DOI:** 10.1038/s41598-024-58199-0

**Published:** 2024-05-01

**Authors:** Serap Gungor, Betul Tosun, Gozde Candir, Nurten Ozen

**Affiliations:** 1 Kahramanmaras Sutcu Imam University, Vocational School of Health Services, Kahramanmaras, Turkey; 2 Hacettepe University, Faculty of Nursing, Ankara, Turkey; 3 Adana City Education and Research Hospital, General Surgery İntensive Care, Adana, Turkey; 4 Demiroglu Bilim University, Florence Nightingale Hospital School of Nursing, Department of English Nursing, İstanbul, Turkey

**Keywords:** Thirst, Oral spray, Pain, Postoperative period, Nurse/nursing, Intensive care units, Health care, Signs and symptoms

## Abstract

This study aims to investigate the effects of intraoral cold water spray on thirst, frequency of oral care and postoperative period pain at surgical incision site in patients having abdominal surgery. The study was carried out as a randomized controlled trial, registered under Clinical Trial Number: NCT05940818. The study involved 110 participants, divided equally into two groups (n = 55): the experimental group and the control group. Data were collected using patient information form, NRS, Intensive Care Oral Care Frequency Assessment Scale (ICOCFAS) and Visual Analog Scale (VAS). The severity of thirst at 1st, 8th, 16th h of post-operative period (p < 0.01) and the frequency of oral care application at 16th h were statistically significantly decreased in the experimental group when compared to the control group (p < 0.01).There wasn’t statistically significant difference between the patients in the experimental and control groups in terms of pain at surgical incision site (p > 0.05). The patient’s thirst and need for frequent oral care in the postoperative period were reduced by the application of a cold water spray. In patients undergoing abdominal surgery, the use of cold water spray application may be recommended to reduce thirst and the need for frequency of oral care application.

## Introduction

While symptoms such as pain, nausea-vomiting and sore throat are focused on in postoperative patients, thirst is considered as an overlooked or less disturbing symptom^1^. Evidence shows that patients experience moderate thirst postoperatively, and thirst is the second most common symptom^1–3^. In studies, the prevalence of thirst in the postoperative period varies between 55.8% and 89.6%^1,4,5^. Typically, an individual experiences thirst when they sense dryness in their oral cavity, throat, and lips, a thickened tongue, an unpleasant and bitter taste in the mouth, and a strong desire to consume water^2,6^. The etiology of postoperative thirst is multifaceted and has not yet been explained in full^1^. Thirst is affected by many factors including individual, environmental, affective, cognitive, social, cultural and health status^2^.

## Background

Thirst is one of the stress factors that decrease the comfort of intensive care patients^7^. When a patient’s oral intake is limited, nurses frequently believe that there is nothing they can do to help them feel less thirsty or dry mouth, and they may not be aware of any techniques. Furthermore, in critical areas of the hospital, thirst is not monitored but neglected^8–10^. It is evident that no evaluation has been made technique routinely used in clinics to evaluate thirst and dry mouth^8,11^. However, thirst is a significant symptom that should be evaluated by the nurse^7^. For thirst management, nurses should evaluate the expressions of thirst and dry mouth of the patients and perform risk assessment with visual controls^12^. Defining the patient’s thirst and making appropriate interventions is an important part of humanistic nursing care^13^.

Numerous potential approaches have been suggested to alleviate thirst, including strategies involving lower temperatures, menthol combined with cold measures, chewing gum, acupressure, early fluid intake, artificial saliva, and the utilization of a thin straw^1^. According to studies, the application of cold can stimulate oral cold receptors in the mouth, specifically within the trigeminal nerve, resulting in heightened salivation and a reduction in the sensation of thirst^14^. Cold water satiates thirst more effectively than body temperature water^15^ and is preferred because it offers greater relief from mouth dryness^16^. Cold water also may stimulate greater saliva production than warm water^17^, which may, in turn, alleviate mouth dryness^18^. Utilizing cold temperatures to alleviate thirst has gained prominence in the field of sensory physiology. This is due to the fact that each sensation hinges on transient receptor potential (TRP) activation. Notably, TRPM8, a receptor responsible for cold thermal transduction, located within oropharyngeal nerve endings, can be activated by low temperatures. The sensation of coldness is transmitted through visceral afferent fibers and afferent nerve fibers from the trigeminal and glossopharyngeal nerves to the somatosensory cortex of the brain. This produces signals of satisfaction with drinking, which relieves thirst^19^. In the postoperative period, it has been claimed that bundle^20^ applications consisting of ice popsicle^21^, mentholated popsicle^2^, oral swab wipes, sterile ice-cold water sprays, and lip moisturizer are effective in thirst management of patients. It was reported in a published systematic review study that cold oral stimuli are effective in reducing postoperative thirst^22^. Patients having abdominal surgery may get thirst relief with the use of cold water, ice, and cold menthol treatments, among other techniques^12,22,23^. Studies have shown that patients can safely use 8–10 ice cubes, equivalent to a total of 40–50 ml (1 ice cube = 5 ml), throughout the day to prevent thirst^1,24^. Due to intubation, blood loss, osmotic imbalance, and extended fasting, surgical patients, and particularly patients undergoing bariatric surgery, are at a high risk of developing thirst^25^ In practice, it is observed that the fasting period of patients undergoing abdominal surgery is longer than recommended in the preoperative and postoperative periods^26,27^. In our country, the use of ice cubes after surgery is not common, and clinics rarely have ice machines for patients. Previous studies have generally attempted to reduce dryness using ice cubes, with room temperature water commonly used in the control group^1,22,28^. Only one study conducted an experimental investigation using a 7–15 °C cold water spray^12^. Our aim was to generate new evidence for nursing practices by planning to apply cold water at a temperature of + 4 °C using a spray, which reduces the risk of aspiration associated with ice cubes. This approach is a response to the lack of evidence and the need for safer alternatives in our clinical setting.

## The STUDY

### Aim

The aim of this study was to examine the results of intraoral cold water spray application on thirst, frequency of oral care application and postoperative pain at surgical incision site in patients undergoing abdominal surgery.

### Research hypotheses

#### H_0_:

 In patients after abdominal surgery, intraoral cold water spray application severity doesn’t affect thirst, frequency of oral care application, and postoperative pain at the surgery site.

#### H_1_:

 In patients after abdominal surgery, intraoral cold water spray application severity lowers thirst, frequency of oral care application, and postoperative pain at the surgery site.

## Methods

### Study design and participants

The study was conducted in the General Surgery Intensive Care Unit of a teaching and research hospital. The study was conducted as a randomized controlled experimental study with two groups. In one group, cold water spray was administered intraorally every hour to patients with a thirst level of 3 out of 10 or more during the hours when oral intake was closed after surgery, while standard nursing care was applied to the other group.

**Figure 1:**
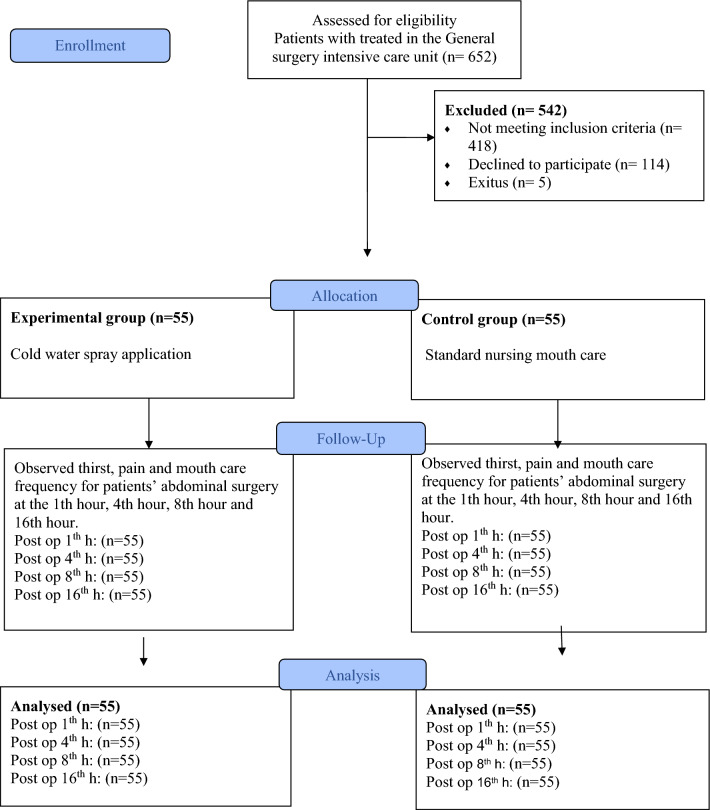
Study flow diagram (CONSORT**)**.

In the clinic, standard oral care is performed by the patient’s nurse every 8 h by wetting the sponge oral care swabs with drinking water without using any solution and is repeated according to the patient’s needs. The CONSORT checklist was used in the study (Fig. 1).

### Inclusion and exclusion criteria

Patients with a level of consciousness of A (Alert level of AVPU) and orientation (place, direction, time), between 18 and 65 years old, with swallowing and coughing reflexes, without nausea and vomiting, without mandibular fracture, without dialysis treatment, with a thirst level of 3 or above a scale from 0 to 10 when asked about thirst^29^, who had been fasting for more than 6 h, whose duration of anesthesia exceeded 1 h, who did not receive mechanical ventilator support, who did not use prosthetic teeth, who underwent elective abdominal surgery, who could communicate well enough to answer postoperative research questions, who could not perform mechanical cleaning of the mouth on their own, and who had no postoperative oral intake for at least 16 h were involved in the study. The study excluded patients who were over 65 years old, had lesions on their oral mucosa and lips, had diseases that hindered communication (such as Alzheimer’s disease or dementia), and had a thirst level of 3 or less before the application of cold spray.

### Sample size

The study sample consisted of patients undergoing abdominal surgery who met the inclusion criteria and agreed to participate in the study. The sample was calculated by predicting a t-test for independent groups. In the study of Serato VM, Fahl-Fonseca L, Birolim MM, Rossetto EG, Mai LD, 2019, the severity of thirst was found as 1.6 ± 1.9 in patients with menthol package (lip hydration and ice popsicles) application and 1.7 ± 1.6 in patients without intervention. Based on this investigation, it was determined that a trial with 90% power and an effect size of 0.6 should consist of 98 patients, 49 in each group. A total of 110 patients (55 in each group) were planned to be incorporated into the study, taking into account potential losses such as exitus, transfer to another unit, or voluntary withdrawal. It was predicted that there would be a 10–15% loss of data (Fig. 1. CONSORT). The study population consisted of 652 patients who underwent surgical operation between April 2022 and March 2023. Among these patients, 418 did not meet the inclusion criteria, 114 patients rejected to participate in the study, 5 patients were re-operated and 5 patients died during the data collection process. The study was completed with a total of 110 patients, including 55 patients in each group.

### Randomization and blinding

Patients to be included in the experimental and control groups were determined by random sampling method with the numbers selected from the random numbers table. A research nurse who was not a part of the research team carried out the randomization and allocation concealment methods in order to minimize potential biases. One of the researchers enrolled and assigned participants to interventions. Blinding of the participants or the researcher was not possible because of distinct visual characteristics of the cold spray.

#### Equipment

Cold Spray bottle: The Cold Spray bottle is a transparent plastic container with a 50 ml capacity. Each press of the spray dispenses 2 ml of liquid. A separate bottle was assigned to each patient, with their name clearly labeled. The bottles were stored in the refrigerator at + 4 °C in the intensive care unit, and the patient’s nurse was informed. The bottles were stored in the refrigerator at + 4 °C in the intensive care unit, and the patient’s nurse was informed. Similar to the study, the temperature of the water used was + 4 °C and approximately 2 ml was sprayed into the mouth^12^.

#### Intervention

The study applied the intensive care oral care frequency assessment scale to 55 patients in the experimental group at the end of the 16th hour. Pain and thirst were also assessed at the 1st, 4th, 8th, and 16th hours postoperatively. For patients with a thirst level of 3 or higher, the bed should be elevated to a 30°–45° degree angle to prevent bronchoaspiration. Cold water, kept in the refrigerator at + 4 °C, should be sprayed into the patient’s mouth three times per hour, starting from the first hour in the postoperative period. Each spray should be approximately 2 ml. This total application of around 6 ml, divided into three doses, is considered safe to prevent respiratory aspiration. A systematic review by Tsai et al.^22^ highlighted the effectiveness of using ice cubes and water volumes ranging from 2 to 30 ml. To alleviate thirst, patients can safely consume 8–10 ice cubes (each equivalent to 5 ml) throughout the day^24,30^. Prior to application, the water was sprayed multiple times into the air to ensure even distribution by removing air from the spout. Following this, the patient was instructed to open their mouth, lift their tongue, and direct the spray towards the sublingual area, as well as the mucosa in both cheeks and on the surface of the tongue. The patient was then asked to keep their mouth closed for 5 min after each application.

The clinic provided standard oral care to patients requiring it. The study recorded the frequency of oral care provided to patients within a 16 h period. The control group did not receive any intervention. The control group was asked the same questions as the experimental group, and their responses were recorded.

### Outcomes

The study primarily focused on evaluating the severity of patients’ thirst and pain. The secondary outcome measured the frequency of oral care needs. In this way, data were collected by the researcher using the Patient Information Form, Numerical Rating Scala (NRS) Thirst, Intensive Care Oral Care Frequency Rating Scale (ICOCFAS) and Visual Comparison Scale.

#### Patient information form

The form, which was prepared by the researchers by utilizing the relevant literature, includes 14 questions regarding the socio-demographic and clinical data of the patients^23,29,31,32^.

#### Visual analogue scale

The Turkish validity and reliability of this scale, which was developed by Price et al.^33^ to evaluate the severity of pain, was performed by Eti Aslan (2004) by evaluating the presence of postoperative pain in patients^33,34^. The postoperative pain at the surgical incision site was graded from 0 to 10 as 0 = no pain and 10 = intolerable pain. Written permission for the use of the scale was obtained from Eti Aslan via e-mail.

#### Numerical rating scala (NRS) thirst

It is a numerical analog scale to be used to objectively assess the patient’s perception of thirst. Thirst perception is scored based on patient self-report from 0 (no thirst) to 10 (intolerable thirst)^1^. For the purpose of analysis, patients were divided into two groups according to their thirst before any intervention: no thirst to mild thirst (0–2 points) and moderate to severe thirst (3–10 points), taking similar studies as an example, although there is no accepted threshold^29^.

#### Intensive care oral care frequency assessment scale (ICOCFAS)

The intensive care oral care frequency assessment scale was developed by Doğu Kökcü & Terzi, (2020) and includes 9 assessment parameters such as patient’s age, lips, teeth, tongue, oral mucosa, salivation, cheeks, nutritional support and respiratory support. Each parameter is scored as 1 = normal, 2 = mild, 3 = moderate, 4 = severe. A minimum score of 9 and a maximum score of 36 can be obtained from the scale. For each condition, such as broad-spectrum antibiotic or steroid treatment, diabetes mellitus diagnosis, low hemoglobin concentration, and immunosuppressive drug use, an additional point was added to the total score. The frequency of oral care was determined based on the total score. The higher total score, correlates with higher frequency of oral care. The Cronbach alpha reliability coefficient of the scale is 0.85^35^. In this study, it was found as 0.75.

### Data collection

**Figure 2:**
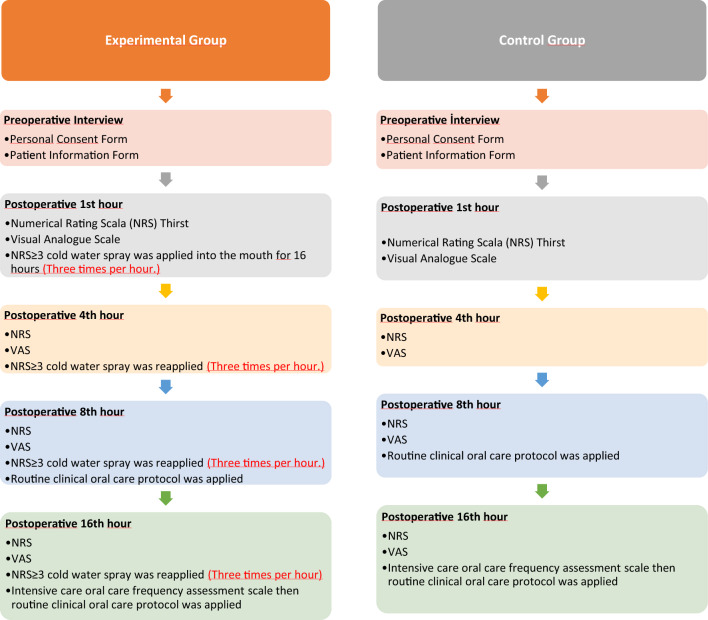
Flow chart.

Data were collected by the researcher through face-to-face interviews with the participants between April 2022 and March 2023. Patients were evaluated in the perioperative period (12 h before intraoperative) and postoperative 1st, 4th, 8th, 16^th^ h in the general surgery intensive care unit. In the experimental group, Interventions were performed by intensive care nurses (Fig. 2).

### Data analysis

The IBM SPSS Statistics for Windows 22.0 (IBM Corp., Armonk, NY, USA). tool was used to analyze the data gathered for this investigation. Number, percentage distributions, mean and standard deviation, and interquartile range (IQR) were used to analyze the data. Chi-square test was used to compare categorical data in the groups. Student-t Test, Mann Whitney-*U* Test, and *Repeated measures ANOVA* were used to analyze the data. A p value of p < 0.05 was accepted as statistically significant.

### Ethical considerations

The required authorizations were acquired from the Hasan Kalyoncu University, Scientific Research Ethical Board in the Faculty of Health Sciences (decision dated 28/02/2022 and numbered 2022/018) and the institution where the study was conducted (decision dated 06/04/2022 and numbered E-96172664-050.06.04) before starting the study. At all stages of the study, the research and publication ethics principles of the Declaration of Helsinki were followed. Between the dates of the study, data related to the study were collected after written informed consent was obtained from patients who met the acceptance criteria and volunteered to participate in the study. The study was registered in the clinical trial database (NCT05940818). Full date of first trial registration is 11.07.2023. The surveys may be used with the consent of the authors who conducted the reliability and validity of the scale via email.

## Results

This study was conducted with a total of 110 patients, including 55 patients in each group (Table 1).

**Table 1:** Comparison of the groups according to some descriptive characteristics at the beginning of the study.

Variable	Control group (n = 55)		Experimental group (n = 55)		Test p
	n	%	n	%	
Gender					
Female	20	36.4	24	43.6	χ^2^ = 0.341
Male	35	63.6	31	56.4	p = 0.560
BMI status					
Underweight (< 18.5 kg/m2)	1	1.8	2	3,6	χ^2^ = 0.033
Normal (18.5–24,9 kg/m2)	15	27.3	14	25.5	p = 0.855
Overweight (≥ 25.0 kg/m2)	39	70.9	39	70.9	
Alcohol use status					
I use	10	18.2	7	12.7	χ^2^ = 0.034*
I Do not use	38	69.1	43	78.2	
I quit	7	12.7	5	9,1	p = 0.854*
Smoking status					
I use	17	30.9	16	29.1	χ^2^ = 0.000
I Do not use	25	45.5	27	49.1	p = 0.1000
I quit	13	23.6	12	21.8	
Chronic disease status					
Yes	27	49.1	35	63.6	χ^2^ = 1.811
No	28	50.9	20	36.4	p = 0.178
Presence of postop NG catheter					
Yes	33	60.0	33	60.0	χ^2^** = **0.000
No	22	40.0	22	40.0	p = 1.000
Amount of fluid given during surgery (ml)	2518.18 ± 1280.05	2000 (1500)	2427.27 ± 939,94	2500 (1000)	z = 1488.00 p = 0.882
Planned fluid intake at postop 24 h	2418.18 ± 550.78	2000 (1000)	2300.00 ± 557,77	2000 (1000)	z = 1334.00 p = 0.242
Preop fasting time (liquid)	13.56 ± 4.31	12 (4)	14.63 ± 9,33	12 (4)	z = 1487.00 p = 0.876
Preop fasting time (solid)	14.90 ± 4.83	14 (4)	17.03 ± 8.97	14 (6)	z = 1255.50 p = 0.119
Duration of surgery	181.36 ± 92.74	150 (60)	183.36 ± 77.10	180 (120)	z = 1431.00 p = 0.622
Age (years)	50.90 ± 14.52	56 (22)	53.20 ± 10.29	56 (18)	z = 1509.00 p = 0.983

Data was presented mean ± standard deviation and median (IQR).

χ^2^ = Chi-square Test, z = Man Whitney *U* Test.

**Figure 3:**
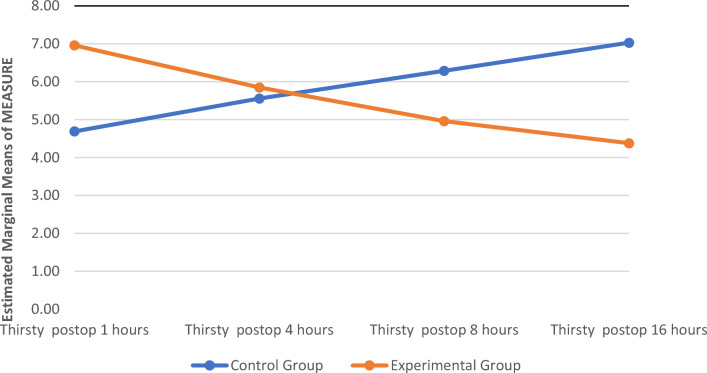
Comparison of the thirsty mean scores of the group receiving standard care and the groups receiving cold water spray.

The severity of thirst in the experimental group was statistically significantly lower than that in the control group at postoperative hours 1, 8, and 16 (p < 0.01). The severity of thirst in the experimental group decreased statistically significantly compared to the control group (${F}_{Grup}=$*108.082,* p: < 0.001) (Table 2) (Fig. 3).

**Table 2:** Comparison of the thirsty mean scores of the group receiving standard care and the groups receiving cold water spray.

Thirsty hours	Control group	Experimental group	Test (z)	p
Postop 1 h	4.69 ± 2.42	6.96 ± 2.19	− 5.153	< 0.001
Postop 4 h	5.56 ± 1.98	5.85 ± 1.89	− 0.785	0.434
Postop 8 h	6.29 ± 2.21	4.96 ± 2.04	3.263	0.001
Postop 16 h	7.03 ± 2.26	4.38 ± 1.77	6.843	< 0.001
Test	${F}_{Grup}=$*108.082*			
p	P = < ,001			

Data was presented mean ± standard deviation.

*F* = Repeated measures ANOVA.

*z* = Man Whitney *U*.

The frequency of oral care for patients in the experimental group, as assessed by the Intensive Care Oral Care Frequency Assessment Scale, was found to be significantly lower than that of the control group (p < 0.01) (Table 3). No intervention-related complications were observed.

**Table 3:** Comparison of the ICOCFAS and pain severity mean scores of the group receiving standard care and the groups receiving cold water spray.

	Control group	Experimental group	Test (z)	p
ICOCFAS^a^	19.27 ± 3.12	15.94 ± 2.62	6.038	0.000
VAS				
Postop 1 h	6.78 ± 2.29	6.74 ± 2.31	0.083	0.934
Postop 4 h	4.69 ± 1.80	4.23 ± 1.92	1.278	0.204
Postop 8 h	3.81 ± 1.70	3.25 ± 1.76	1.705	0.091
Postop 16 h	2.89 ± 1.74	2.69 ± 1.71	0.605	0.547
Test	${F}_{Grup}=$*3.347*			
p	p = 0.070			

*ICOCFAS* intensive care oral care frequency assessment scale, *VAS* visual analogue scale was used to assessed the pain.

^a^Postop 16 h.

When the level of postoperative pain at the surgical incision site was compared, no statistically significant difference was found between the experimental and control groups at postoperative 1, 4, 8, and 16 h (p > 0.05). There was no difference between the experimental group and the control group in terms of the postoperative pain at the surgical incision site (Group = 3.347, p = 0.070) (Table 3).

## Discussion

It is known that thirst is a disturbing symptom for patients in the postoperative period^1,2^. However, thirst is one of the most common complaints expressed by critically ill patients since oral water intake is often not possible. Treatment of thirst typically involves oral fluid intake. Managing the patient’s thirst and providing oral care are significant aspects of symptom management^13^. In this study, it was discovered that the use of cold water spray into the mouth every hour in the postoperative period to relieve thirst in patients undergoing abdominal surgery reduced the thirst symptom in the 1st, 8th and 16th hours postoperatively. When the literature is examined, thirst preventive methods have been tried in many studies and positive results have been obtained^36,37^. It has been reported that mentholated cold water, psyllium^36^, mentholated popsicle^37^, 0.75% citric acid spray^29^, and ice cream^28^ reduce thirst in intensive care unit patients after surgery. As a result of cold water spray that was applied to patients undergoing major abdominal surgery, it was observed that the severity of thirst decreased after the 8th postoperative hour^12^. Zimmerman and colleagues identified the neural pathway that regulates thirst through oropharyngeal stimulus. They observed that thirst-promoting neurons were rapidly inhibited with the consumption of water, and drinking ceased once neuronal activity returned to baseline. This inhibition occurred before any changes in plasma osmolarity, indicating that thirst was quenched before the water was absorbed into the bloodstream. The inhibition was found to be temperature-dependent, with cold water promoting a more rapid decrease in neuron activity. It occurred when the oral cavity was cooled by the application of cold metal. Temperature is a crucial factor in inhibiting thirst-promoting neurons. Cold water ingestion leads to a more rapid decrease in neuronal activity, resulting in faster thirst quenching^38^. This may not explain why patients experience profound thirst even when clinically hydrated but does provide a compelling explanation for why the oral application of water and ice chips is effective at quenching thirst, even without the oral ingestion of enough water to affect blood tonicity^11,13,39,40^. It was found that the utilization of ice water oral swab and menthol lip moisturizer in the intensive care unit reduced the intensity of thirst in adult patients^11^. Based on various studies conducted using different products and methods, it has been concluded that cold applications can reduce thirst. It is important to note that this conclusion is based on objective evaluations and not subjective opinions. This is due to the fact that the sprayed water is cold and affects many areas in the mouth, stimulating cold receptors and providing a physiological effect.

The study found that patients who received cold water spray application required less frequent oral care compared to those who received standard oral care. Another study on thirst perception and dry mouth in intensive care patients showed that oral care provided temporary relief for only one hour and had no lasting effect. Therefore, it was suggested that patients require more frequent oral care^13^. Planned oral care was found to be effective in reducing thirst intensity and dry mouth in patients in the intensive care unit^31^. It has been reported that ice water oral swabs and lip moisturizer with menthol are effective in alleviating thirst and dry mouth in patients in the intensive care unit^11^. The administration of a bundle consisting of vitamin C sprays, mint water mouthwash, and lip moisturizer to critically ill patients resulted in a reduction in thirst intensity and an improvement in the condition of the oral mucosa^41^. After applying frozen saline and providing routine oral care to critically ill patients following surgery, it was observed in the surgical intensive care unit that the patients experienced a reduction in dry mouth and an improvement in the condition of their lips, tongue, mucosa, and saliva^32^. Appropriate interventions that evaluate the thirst of patients can help reduce the frequency of oral care by keeping the mucosa moist and clean.

Acute pain is a frequent issue in surgical clinics. To manage pain, early enteral nutrition and mobilization are recommended to prevent postoperative loss of function and associated morbidities^26^. In this study, both groups experienced a decrease in postoperative pain at the surgical incision site. However, the application of cold water spray did not result in a significant difference in postoperative pain at the surgical incision site. Another study investigated the impact of oral water and ice application in managing early postoperative thirst. The study found that patients in the ice application group had lower pain score values compared to those in the water group^42^. Early initiation of oral intake in the postoperative period can accelerate the patient’s recovery, reduce pain, and shorten hospitalization duration and costs^43^. Research has shown that shortening the fasting period before colorectal surgery can significantly reduce anxiety and pain, as well as support the recovery of gastrointestinal function in patients^44^. It has been observed that shortening preoperative fasting with carbohydrate and protein-containing liquids before gynecologic surgeries reduces thirst, hunger, pain, and agitation^45^. In a study on the experiences of patients with preoperative nutrient/fluid restriction and the knowledge and practices of nurses on this subject, a weak positive relationship was found between the patients’ postoperative headache score and thirst score^46^. These results suggest that fasting and dehydration may cause undesirable outcomes such as postoperative pain at the surgical incision site, but since pain can be influenced by a variety of factors, this study did not provide a significant effect.

### Strengths and limitations

Being the first study to date to assess the impact of intraoral cold water spray treatment on oral care frequency and pain at the incision site in patients after elective abdominal surgery is one of the study’s strengths.

The study has limitations that require consideration. Firstly, it is limited to patients undergoing elective abdominal surgery in the intensive care unit, which may limit the generalizability of findings to a broader population. Additionally, blinding was challenging due to the nature of the intervention (cold spray), making it impossible to blind both participants and researchers. The study’s conclusions regarding pain management may have been affected by the complexity of postoperative pain, which is influenced by various factors and may not have been entirely controlled by the intervention. Additionally, the sample size and diversity were limited to a single center setting, which raises concerns about the representation of a wider range of patient demographics.

## Conclusions

Based on the study’s findings, the controlled application of cold water spray to intensive care patients during the recovery phase reduced their thirst experience and the frequency of required oral care. Intensive care nurses need to be made aware that applying a small amount of cold water during periods of oral intake cessation does not cause complications in patients. Further studies with different patient groups are necessary.

## Relevance to clinical practice

It is seen that thirst, which is ignored in the postoperative period, is a disturbing and significant symptom of patients. Nurses should know the methods used by monitoring thirst in order to provide better care to patients and to use proven practices.

In this study, it was found that giving abdominal surgery patients a cold water spray throughout the recovery phase reduced their perception of thirst and their need for oral care.

For nurses, using cold water spray application is a useful and affordable technique.

## Data Availability

The raw patient-level data supporting the conclusions of this article will be made available by the author Serap GUNGOR’s (e-mail: serap_32_06@hotmail.com ) upon request.
